# Diagnostic Maze: Malignant Phyllodes Tumor Mimicking Metaplastic Breast Carcinoma

**DOI:** 10.7759/cureus.62114

**Published:** 2024-06-10

**Authors:** MonishaRita Jayaraman, Lakshmipriya V, Nithin Diwagar, Muthuvel Esakki

**Affiliations:** 1 Pathology, Saveetha Medical College and Hospital, Saveetha Institute of Medical and Technical Sciences, Saveetha University, Chennai, IND

**Keywords:** heterologous mesenchymal differentiation, malignant phyllodes tumour, spindle cell lesion, immunohistochemistry staining, phyllodes tumors

## Abstract

Phyllodes tumors (PTs) are uncommon breast tumors. They represent a spectrum of benign to malignant neoplasms. These erratic tumors have traits ranging from fibroadenomas on one end of the spectrum to sarcomas on the other end. The presentation of PT is variable, thus posing a diagnostic difficulty. Malignant PTs are often associated with recurrence, poor prognosis, and adverse disease outcomes. The recent genetic studies produced by genome sequencing offer information about the molecular pathophysiology of PT, aid in enhancing diagnostic precision, and suggest possible therapeutic targets in cases of malignant PT. Planning a treatment modality and prognostication requires meticulous histopathological sampling, as it relies on a correct histopathological diagnosis. There are no definitive guidelines for surgical management and targeted therapy for malignant PTs due to the rarity of these cases and very little available literature on these topics. Here in this article, we address a malignant PT in a 74-year-old female that presented as a breast mass mimicking metaplastic breast carcinoma histologically. This article also illuminates how immunohistochemistry plays a vital role in the diagnosis of this tumor.

## Introduction

Phyllodes tumor (PT) of the breast is a rare biphasic fibroepithelial neoplasm. Malignant PT is more common in Asian women. The incidence of PT is relatively higher in Asian women and is around 6.92%, as compared to the Western population, where the incidence is 0.3 to 1.5% [1-3]. PTs account for less than 1% of the breast tumors [2]. Studies show that PT comprises 0.3% to 1% of all breast tumors [3]. Muller, in 1838, introduced the term cystosarcoma phyllodes. It is derived from the Greek words phyllon, which means leaf-like, and sarcoma, which means fleshy appearance. The majority of PTs are benign, and hence the name sarcoma can be misleading [2]. PT is a firm to hard, bulging mass that is well-defined with a cut surface showing a tan-pink to grey-white color [1,2]. Large lesions resemble leaf buds with their whorled appearance and clefts. In large lesions, variable amounts of necrosis and hemorrhage are seen [1]. A pathologist must collect sufficient representative sections during grossing, which is at least one section for every centimeter of the tumor [1]. Many studies show evidence that fibroadenoma and PTs have a common origin [3,4]. Interactions between epithelium and stroma are considered to be the well-suited pathogenesis for the development of PTs [3-5]. Recent studies in the molecular pathogenesis of PTs show that Mediator Complex Subunit 12 (MED12) somatic mutation is often recognized in both fibroadenomas and PTs, thus proving that they both have a common origin [3]. This article focuses on the diagnostic hurdles encountered while reporting a malignant PT in routine practice and ruling out metaplastic breast carcinoma (MpBC), which is a close differential diagnosis.

## Case presentation

**Figure 1:**
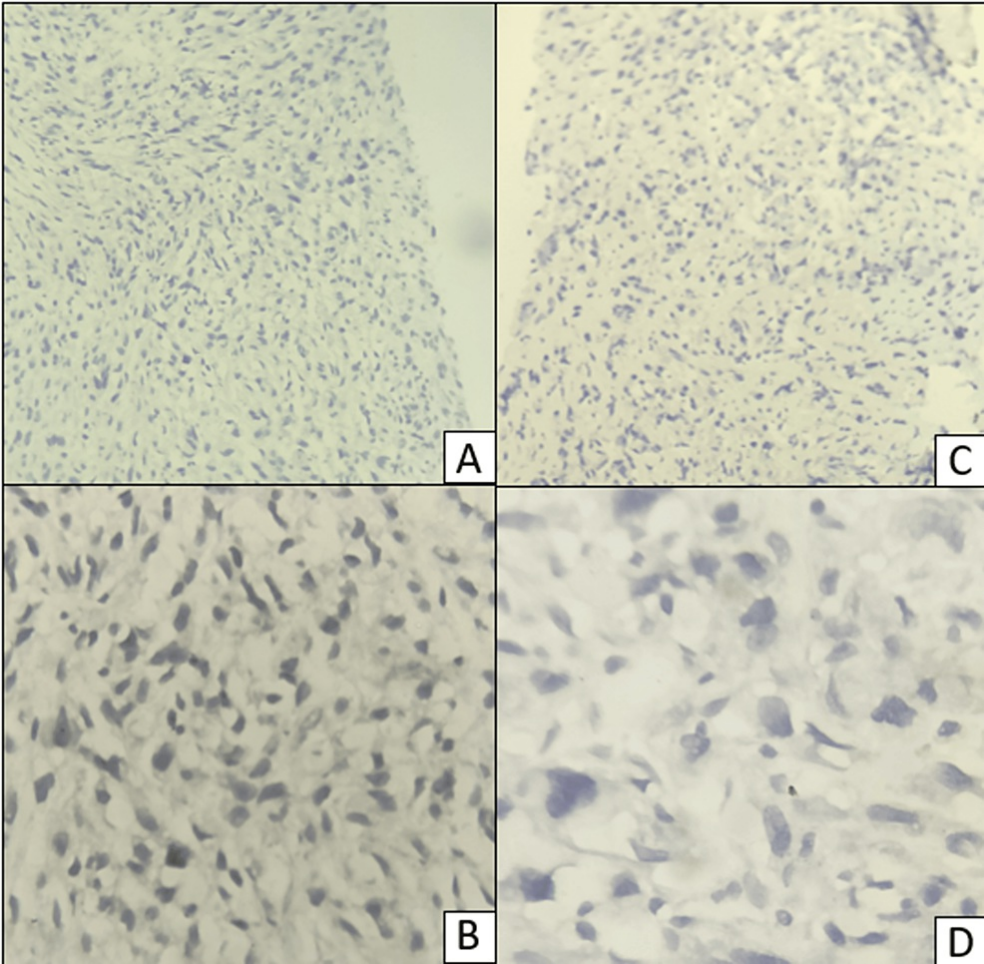
An immunohistochemical (IHC) stained section of the breast mass showing negative expression of the estrogen receptor (A and B) and progesterone receptor (C and D). A and C: ×100; B and D: ×400. (A) 10× view and (B) 40× view, both negative for ER; (C) 10× view and (D) 40× view, both negative for PR. Negative indicates that the nucleus did not take up the brown color of the diaminobenzidine (DAB) stain.

**Figure 2:**
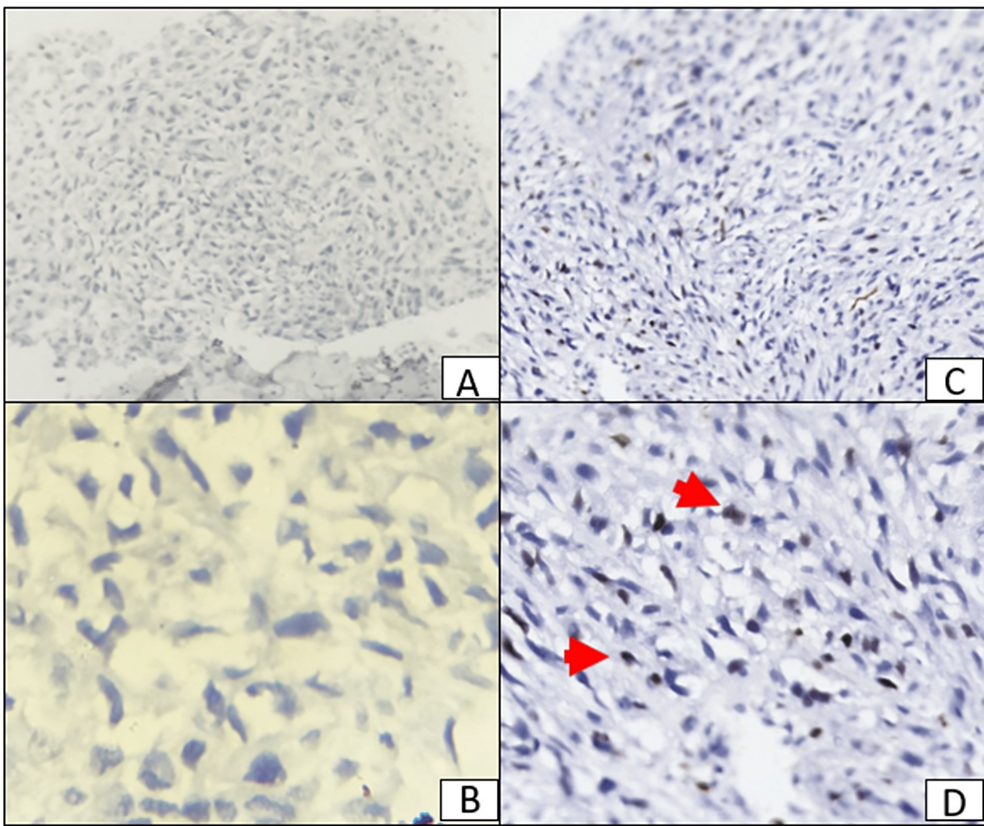
An immunohistochemical (IHC) stained section of the breast mass showing negative expression of the Her2neu (A and B) and positive expression (red arrows) of the Ki67 in more than 10% of the cells (C and D). A and C: ×100; B and D: ×400. (A) 10× power view and (B) 40× view of negative staining for Her2neu; (C) 10× power view and (D) 40× view of Ki67 positive staining in more than 10% of the cells. Positivity is indicated by red arrows in D.

A 74-year-old female presented to the outpatient department with complaints of pain in the right breast, which was insidious in onset and gradual in progression. The pain was constant and non-radiating, with no aggravating or relieving factors. There were no complaints of nipple discharge, association of pain with menstrual cycle, loss of appetite, or loss of weight. On examination, there was a mass in the right breast. A solitary lump of 10 × 7 cm in the right upper outer quadrant, ill-defined margins, uneven surface, and firm to hard in consistency. The swelling moved with the breast tissue and was not fixed to the chest wall or pectoralis minor. There were no palpable axillary lymph nodes. The skin over the swelling was unremarkable, and there was no nipple retraction. A tru-cut biopsy of the mass was done, and a histopathology report was given as invasive ductal carcinoma of no specific type grade 2. Immunohistochemical investigations were done for estrogen receptor (ER) (Figures 1A-1B), progesterone receptor (PR) (Figures 1C-1D), Her2neu (Figures 2A-2B), and Ki67 (Figures 2C-2D). Er, PR, and Her2neu were all negative, and the Ki67 labeling index showed positivity in more than 10% of the tumor cells.

**Figure 3:**
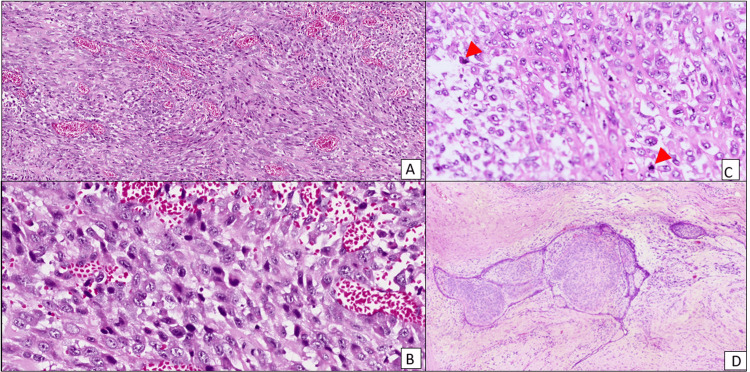
Hematoxylin and eosin-stained section of the breast mass showing sheets of pleomorphic spindled cells (A), with pleomorphic hyperchromatic nuclei (B) and few atypical mitoses (C, red arrows), associated with hypercellular stromal overgrowth (D). A and D: ×100, B and C: ×400. (A) Sheets of spindle cells. (B) Malignant pleomorphic cells with hyperchromatic nuclei. (C) Atypical mitosis in the tumor is indicated by red arrows. (D) Stromal overgrowth.

**Figure 4:**
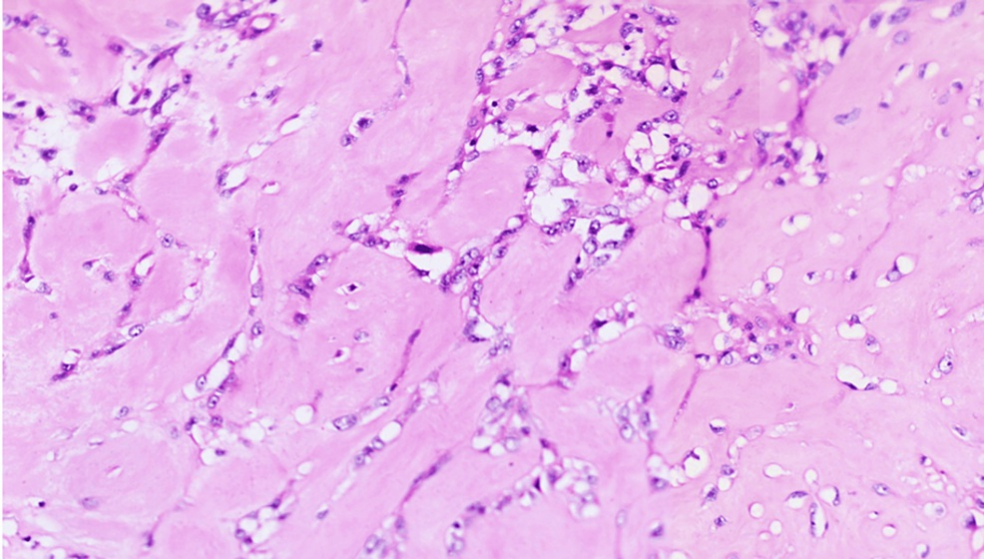
Hematoxylin and eosin-stained section of the breast mass showing extensive areas of osteoid differentiation (×400).

**Figure 5:**
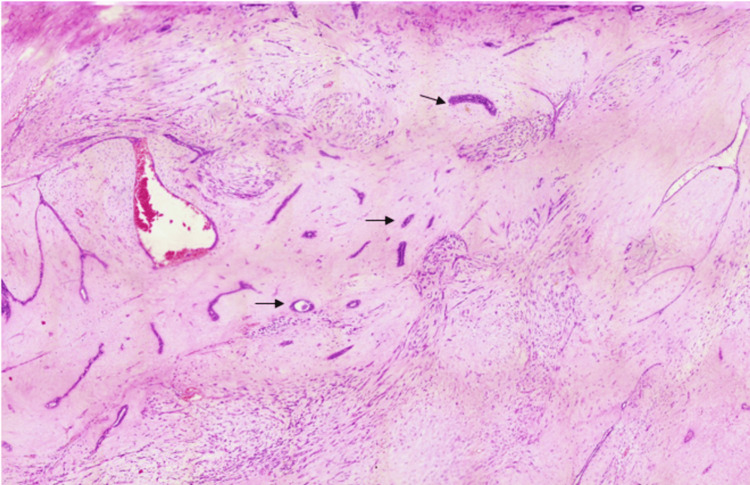
Hematoxylin and eosin-stained section of the breast mass showing benign ducts (×100).

**Figure 6:**
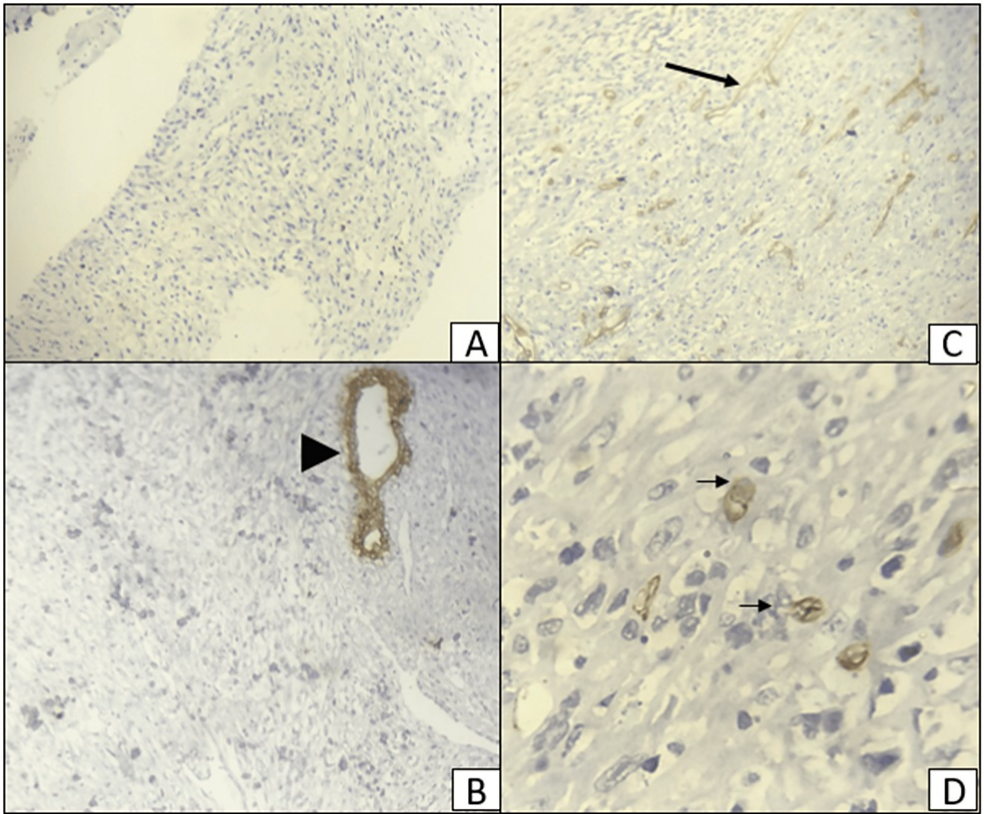
An immunohistochemical (IHC) stained section of the breast mass showing negative expression of the cytokeratin (CK) (A and B) and focal positive expression (black arrows) of CD34 (C and D). A and C: ×100, B and D: ×400. (A) 10× power view and (B) 40× view of negative staining for CK, with positive internal control (ductal epithelium) indicated by a black arrowhead. (C) 10× power view and (D) 40× view of CD34 focal positivity in a few cells, with positivity shown by black arrows in D.

The patient underwent surgery, and right modified radical mastectomy (MRM) with axillary lymph node dissection was done. Following that, the MRM specimen, measuring 6 × 4.3 × 4 cm, was submitted for histopathological processing and investigation. The specimen was thoroughly examined and sampled. Microscopy showed sheets of pleomorphic spindle cells with hyperchromatic nuclei with more than 10 mitoses per 10 high-power fields (hpf) (Figure 3). There were extensive areas of osteoid differentiation (Figure 4). A few benign ducts were seen within the lesion, and the features suggest malignant PT (Figure 5). Immunohistochemical studies were done to rule out MpBC since the latter can also exhibit features of heterologous differentiation. Cytokeratin (CK) was done and was found to be negative (Figure 6). CD34 was done and appeared to be focally positive. The microscopy and immunohistochemistry were consistent with malignant PT.

## Discussion

PT of the breast is a fibroepithelial neoplasm composed of intracanalicular growth pattern and stromal hypercellularity [1,2]. They almost always have cleft-like spaces lined by epithelium. PTs are classified into benign, borderline, and malignant, according to WHO. PTs can be histologically heterogeneous, and the presence of a foci with benign, borderline, and malignant features can be found in the same neoplasm [2,3]. Thus, thorough gross examination and sampling are necessary to grade a tumor. Various pathological features are taken into consideration to classify a neoplasm as benign, borderline, or malignant. These features include stromal cellularity, stromal overgrowth, stromal atypia, mitoses, and tumor margins [4,5]. To call a PT malignant, the tumor should have marked stromal hypercellularity, marked stromal atypia, stromal overgrowth, more than nine mitoses per 10 high power fields (>9/10 hpf), and infiltrative tumor margins [4-6]. A PT is considered malignant if there is the presence of heterologous elements like osteosarcoma or chondrosarcoma [4-6]. The metaplastic changes of the malignant stromal cells are said to be the cause of heterogeneity in malignant PTs [7,8]. This makes it a potential differential diagnosis to MpBC and sarcomas.

A heterogeneous group of invasive breast cancers known as metaplastic carcinomas are distinguished by their neoplastic epithelium differentiate into squamous cells and/or mesenchymal-like components such as chondroid, osseous, and spindle cells [9]. Less than 1% of invasive breast tumors are MpBC. Postmenopausal women are more likely to develop MpBC, which usually is negative for hormone receptors like ER and PR [9,10]. There are various immunohistochemical markers to differentiate between malignant PT and MpBC. A panel of CKs like CKAE1/AE3, CK5/6, 34bE12, cam 5.2, and myoepithelial marker p63 can be used for differentiating as they all show variable staining patterns with MpBC but often negative in malignant PT. CD34, which is a glycosylated transmembrane protein and a marker of vascular endothelial cells, can be used here. CD34 is negative in MpBC but positive in up to 75% of the cases of malignant PTs [4,10-12]. When the diagnosis is uncertain even after histopathological and immunohistochemical studies, several studies show that it is better to report it as a malignant spindle cell neoplasm with a detailed description of histopathological features and to recommend surgical excision [12,13]. It is absolutely necessary to recommend surgical excision because the treatment varies in each of the cases. In the case of malignant PT, sentinel lymph node biopsy or lymph node dissection is not suggested as hematogenous spread is more common and spread through lymphatics is extremely rare [5,11-13]. Adjuvant radiotherapy and adjuvant or neoadjuvant chemotherapy, which is a better-suited management for MpBC, are not recommended for malignant PT as their role here remains uncertain.

Malignant PTs are found to have mutations in the telomerase reverse transcriptase (TERT) gene [7]. TERT mutation results in high levels of TERT mRNA due to TERT amplification in these cases [7-9,13]. The other mutation of interest is in MED12, which is also seen in fibroadenoma, uterine leiomyomas, and leiomyosarcomas. Studies have shown that MED12 mutation affects the growth of smooth muscles, especially in the estrogen- and progesterone-dependent tissues [7,13]. Some other studies investigated p53, Ki67, CD117, EGFR, p16, and VEGF for the histological grading of malignant PT, but they were proved not to be clinically useful [3,10]. There are many ongoing studies due to the promise of molecular science and ongoing research that might help to predict the behavior of PTs. A few parameters like microvessel density, factor XIIIa stromal positivity, and proliferative activity utilizing MIB-1 and S-phase fraction are thought to be possibly beneficial in the prediction of progression of PTs [8,11]. The most investigated is probably p53, where some writers have proposed that it may have prognostic significance but not enough evidence is available in the literature. More recent studies have discovered a link between malignant PT and stromal overexpression of c-myc and c-kit [12-14]. Consequently, it is crucial that we continue to study its genetic makeup since it allows for the development of more precisely targeted treatment options for these kinds of tumors.

## Conclusions

This article focuses on the dilemma faced during the reporting of a breast tumor with heterologous elements. Malignant PTs should be included in the list of differential diagnoses along with sarcomas, when encountering lesions of the breast with sarcomatous differentiation. It is absolutely essential to differentiate between the two due to the differences in their management protocols, and also, early diagnosis is associated with a better prognosis. It is evident that a final diagnosis of this entity cannot be made on a tru-cut biopsy and thus warrants an excision biopsy. Large case series and long-term follow-up of MPT patients with heterologous sarcomatous features are needed for future research in order to anticipate biological behavior, prognostic variables, and recommendations for targeted therapy.
